# Fibrillar β-amyloid 1-42 alters cytokine secretion, cholinergic signalling and neuronal differentiation

**DOI:** 10.1111/jcmm.12343

**Published:** 2014-08-11

**Authors:** Linn Malmsten, Swetha Vijayaraghavan, Outi Hovatta, Amelia Marutle, Taher Darreh-Shori

**Affiliations:** a Karolinska Institutet, Department of Neurobiology, Care Sciences and Society, Center for Alzheimer Research, Division of Translational Alzheimer NeurobiologyStockholm, Sweden; b Department of Clinical Science, Intervention and Technology, K54 Karolinska University Hospital HuddingeStockholm, Sweden

**Keywords:** human microglia, Alzheimer's disease, inflammation, neurospheres, human embryonic stem cells, neurogenesis, gliogenesis

## Abstract

Adult neurogenesis is impaired by inflammatory processes, which are linked to altered cholinergic signalling and cognitive decline in Alzheimer's disease. In this study, we investigated how amyloid beta (Aβ)-evoked inflammatory responses affect the generation of new neurons from human embryonic stem (hES) cells and the role of cholinergic signalling in regulating this process. The hES were cultured as neurospheres and exposed to fibrillar and oligomeric Aβ_1-42_ (Aβf, AβO) or to conditioned medium from human primary microglia activated with either Aβ_1-42_ or lipopolysaccharide. The neurospheres were differentiated for 29 days *in vitro* and the resulting neuronal or glial phenotypes were thereafter assessed. Secretion of cytokines and the enzymes acetylcholinesterase (AChE), butyrylcholinesterase (BuChE) and choline acetyltransferase (ChAT) involved in cholinergic signalling was measured in medium throughout the differentiation. We report that differentiating neurospheres released various cytokines, and exposure to Aβf, but not AβO, increased the secretion of IL-6, IL-1β and IL-2. Aβf also influenced the levels of AChE, BuChE and ChAT in favour of a low level of acetylcholine. These changes were linked to an altered secretion pattern of cytokines. A different pattern was observed in microglia activated by Aβf, demonstrating decreased secretion of TNF-α, IL-1β and IL-2 relative to untreated cells. Subsequent exposure of differentiating neurospheres to Aβf or to microglia-conditioned medium decreased neuronal differentiation and increased glial differentiation. We suggest that a basal physiological secretion of cytokines is involved in shaping the differentiation of neurospheres and that Aβf decreases neurogenesis by promoting a microenvironment favouring hypo-cholinergic signalling and gliogenesis.

## Introduction

Inflammation in neurodegeneration is associated with progressive dysfunction and loss of neurons and may play a causative or propagating role during the course of Alzheimer's disease (AD). Inflammation is conceivably mediated by the chronic inflammatory responses following the accumulation of amyloid-β (Aβ) peptides in the AD brain [1], as deduced from observations of increased astrogliosis and microgliosis in the vicinity of Aβ plaques [2]. The production of small soluble aggregates of Aβ peptides, known as Aβ oligomers (AβO), induce neurotoxic effects in the absence of Aβ plaques in *in vivo* studies, and may be an early event in the pathogenesis of AD [3,4]. Both AβO and insoluble fibrillar Aβ (Aβf) aggregates, which assemble in the neuritic plaques in the AD brain, activate microglia, leading to exaggerated expression and release of inflammatory cytokines and chemokines with consequential neurodegenerative effects [5,6].

Microglia act as native immune cells with phagocytic functions in the brain. In contrast to their putative role in neurodegeneration, microglia may also promote neuroprotective mechanisms in the brain through phagocytosis of Aβ and release of neurotrophic factors [7,8]. Microglia are also implicated in non-neural regulation of postnatal neurogenesis and neuronal development [9].

A link between inflammation and impairment of neurogenesis and cognitive decline during aging and AD has been proposed [10,11]. Earlier findings have suggested that cholinergic signalling in the central nervous system (CNS) has anti-inflammatory effects, whereby the neurotransmitter acetylcholine (ACh) inhibits cytokine release by acting on α7 nicotinic ACh receptors (nAChRs) expressed on macrophages [12]. Furthermore, it has been suggested that Aβ accumulation disrupts normal inflammatory regulation in the brain by increasing the activity of the ACh-hydrolysing enzyme butyrylcholinesterase (BuChE) [13–16]. Recently, we demonstrated that both astrocytes and stem cells secrete the ACh synthesizing enzyme choline acetyltransferase (ChAT), and suggested that the physiological function of extracellular ChAT is to maintain steady-state equilibrium of hydrolysis and synthesis of ACh [17]. We thus hypothesize that excessive extracellular accumulation of Aβ may cause imbalances in cholinergic signalling, which facilitates increased degradation of ACh and enhanced cytokine expression and release. Such an exaggerated inflammatory microenvironment could in turn affect the neurogenic niche in the brain by altering the migration, proliferation and differentiation of endogenous neural stem cells. We have previously shown that AβO_1-42_ disrupts the differentiation of human embryonic stem (hES) cell-derived neurospheres into functional neurons, while Aβf_1-42_ promotes gliogenesis *in vitro* [18]. We propose that altered cholinergic signalling and related changes in cytokine secretion is one of the mechanisms underlying impaired neuronal differentiation of neural progenitors following Aβ exposure.

However, the impact of inflammatory processes on neurogenesis is difficult to study in the brain of AD patients. Although transgenic mice and rats do mimic some crucial features of the pathogenesis observed in AD brain and can provide models for studying *in vivo* regenerative processes in an AD-like environment, there are differences between developmental signals, gene expression profiles and growth factor requirements for rodents and humans [19–22]. These discrepancies could be mitigated by the use of human cell lines.

In the current study, we investigated whether Aβ-induced changes on neural differentiation involve alteration of inflammatory events, such as cytokine secretion and microglial activity. Stem cells have been shown to be able to respond to cytokine signalling [23,24] and may have a close cross-talk with immune competent cells, thereby proper adjustment of their proximate microenvironment [25]. Furthermore, human neural stem/progenitor cells derived from embryonic stem cells and foetal brain have been shown to secrete the cytokines IL-10 and TGF-β [26].

In this study, differentiating neurospheres derived from hES cells and human primary microglia cultures were exposed to Aβf_1-42_ or AβO_1-42_ peptides to study the secretion profile of inflammatory cytokines, cholinergic signalling and neural differentiation. Insights into these processes are critical for the development of new treatment strategies aiming to curtail or modulate pathogenic immune responses that affect regenerative processes in AD.

## Materials and methods

### Human embryonic stemcell and neurosphere cultures

Two fully characterized hES cell lines, HS293 and HS346, were used in this study and details regarding their derivation and characterization have been described in previous publications [27–29]. Both lines were derived from donated supernumerary blastocyst-stage embryos obtained after informed consent, with approval from the Ethics Board at the Karolinska Institute, Sweden. These hES lines have remained chromosomally stable after many (>100) passages; HS293 has a karyotype 46, XY and HS346 46, XX.

For neural induction, colonies of HS293 and HS346 cells grown on human foreskin fibroblasts were subsequently removed from the feeder layer, and the cultures were expanded in serum-free DMEM/F12+ glutamax medium supplemented with B27 (1:50) heparin (5 μg/ml), antibiotic-antimycotic mixture (1:100) and EGF + bFGF (20 ng/ml each, Sigma-Aldrich, St. Louis, MO, USA) at 37°C in a 5% CO_2_ humidified incubation chamber.

Differentiation experiments were carried out by plating 2–3 neurospheres/well in 6-well tissue culture plates on poly-D-lysine- and laminin-coated cover slips, which is referred to as day 0 in this study. The cells were subsequently cultured for 27–29 days of differentiation. Neurospheres were differentiated following a stepwise protocol: culture in neuronal induction medium consisting of DMEM/F12+ glutamax: Neurobasal medium, B27 (without vitamin A; 1:50) and N2 supplement (1:100) for 4 days. From day 4, neural proliferation medium (NPM) consisting of DMEM/F12+ glutamax: Neurobasal medium, N2 supplement (1:200), B27 (1:100) and bFGF (20 ng/ml) was added as we have previously described in detail [18,21,30]. The medium was replaced twice every week during differentiation, and AβO_1-42_, Aβf_1-42_ (100 nM) (rPeptide, Bogart, GA, USA) or conditioned medium (CM) from microglia treated with AβO-CM (100 nM), Aβf-CM (100 nM), control-CM (1:2; without Aβ) or LPS-CM (100 ng/ml) (100 ng/ml; Sigma-Aldrich) were added once a week. All media and cell culture reagents were purchased from Invitrogen (Carlsbad, CA, USA) unless otherwise stated. Both HS293 and HS346 cells were used in each of the experiments performed; no differences were observed between these cell lines.

After 72–96 hrs of incubation, all cultured media were carefully collected for ELISA measurements, and were immediately replaced for all the cultured wells, irrespective of treatment. This procedure should effectively eliminate any differences, which would otherwise occur if the incubation time were different between the treatments. The only exception was the medium at 1 day of differentiation (1 DOD), which was added to the cells 24 hrs prior to the collection. All the collected media were kept frozen in smaller proportions until the enzymatic and ELISA assays.

As another precautionary step and to correct for differences that may arise by the number of cells in each neurospheres as well as the number of cultured neurospheres in each culture well, the individual collected medium from each well at 11 DOD was considered as the baseline sample and the values from the subsequent DOD were normalized to this baseline. All related samples were analysed at the same time and on the same microtiter plate. This was then repeated in a total of three individual experiments by using neurospheres from different passages.

### Human microglia cell cultures

Three pure populations of primary microglia cells (from three different donors) originally derived from human foetal brain tissue were purchased and cultured according to the manufacturer's instructions (3H Biomedical AB, Uppsala, Sweden). Briefly, microglia were cultured on poly-l-lysine-coated 24-well plates at a density of 1.3 × 10^5^ cells/well in DMEM/F12+ glutamax medium supplemented with 5% foetal bovine serum and 10 ng/ml recombinant human macrophage colony-stimulating factor (3H Biomedical AB). The CM was obtained by exposing the microglia to AβO_1-42_ or Aβf_1-42_ (100 nM) or lipopolysaccharide (LPS) in the medium described above. The CM was collected from the microglia cells 48 hrs later, briefly centrifuged and stored at −20°C until used. The CM samples were labelled according to the respective treatment: control-CM (for untreated microglia), AβO-CM (100 nM), Aβf-CM (100 nM) or LPS-CM (100 ng/ml). All medium and cell culture reagents were purchased from Invitrogen unless otherwise stated.

### Aβ preparation

Aβf_1-42_ and AβO_1-42_ preparations were made as described previously [18]. Briefly, Aβf aggregates were prepared by dissolving NaOH-pre-treated recombinant Aβ_1-42_ (rPeptides, Bogart, GA, USA) in PBS and incubating with gentle shaking for 72 hrs at 37°C. AβO was prepared by dissolving 1,1,1,3,3,3-hexafluoro-2-propanol-pre-treated recombinant Aβ_1-42_ (rPeptides) in DMSO, followed by sonication and filtration. Aliquots were then stored at −80°C until required.

### Cell proliferation and cell viability assays

For the proliferation and viability assays, neurospheres were dissociated by using TrypLE (Invitrogen) for 5 min. at 37°C. The cells were plated in a 96-well tissue culture plate (10,000 cells/well) 24 hrs prior to the administration of AβO_1-42_ (1 pM–1 μM) or Aβf_1-42_ (1 pM–1 μM) or control-CM, AβO-CM, Aβf-CM or LPS-CM. The extent of cell proliferation was measured 5 days after treatment by using a bromodeoxyuridine (5-bromo-2-deoxyuridine, BrdU) incorporation assay according to the manufacturer's instructions (Roche, Mannheim, Germany). Cell viability was assessed 5 days after treatment by using a CellTiter 96 AQ_ueous_ One Solution assay (Promega, Madison, WI, USA) according to the manufacturer's instructions.

### Immunocytochemistry

Microglia cultured for 14 days on poly-l-lysine-coated cover slips were fixed with 4% paraformaldehyde at 4°C for 20 min., made permeable in blocking buffer (3% normal donkey serum in PBS containing 0.05% Triton X-100) and incubated overnight at 4°C with goat polyclonal anti-ionized calcium-binding adaptor molecule-1 (IBA-1, 1:100; Abcam, Cambridge, UK) antibody. Following three 5-min. washes in PBS, secondary antibody conjugated with Alexa Fluor (AF) 546 donkey anti-goat (1:500; Molecular Probes, Eugene, OR, USA) were added for an 1-hr incubation period at room temperature in the dark.

The proportion of neurons and astroglia in differentiated neurospheres was assessed by fluorescent immunocytochemistry, essentially as described in Wicklund *et al*. [18]. Incubation with the primary antibodies mouse monoclonal anti-β-tubulin III (1:250; Sigma-Aldrich), mouse monoclonal anti-microtubule-associated protein 2 (MAP2 2a+2b; 1:250; Sigma-Aldrich), rabbit polyclonal anti-MAP2 (1:250; Millipore, Temecula, CA, USA) and rabbit polyclonal anti-human glial fibrillary acidic protein (GFAP; 1:250; Dako Cytomation, Glostrup, Denmark) was performed, followed by incubations with secondary antibodies conjugated with AF 546 donkey anti-mouse or AF 488 donkey anti-rabbit (1:500; Molecular Probes). The results were quantified by counting the number of β-tubulin III/MAP2/GFAP-immunoreactive cells co-labelled with 4′,6-diamidino-2-phenylindole in three to six random fields for each experiment (>600 cells counted) under a Nikon E800 microscope at 20× magnification.

### ELISA measurements

To control for endogenous Aβ release as well as the remaining Aβ levels in control-CM and Aβf-CM, the concentration of Aβ was quantified by a human Aβ_1-42_ sandwich ELISA kit (Invitrogen, Camarillo, CA, USA), according to the manufacturer's instructions. The Aβ_1-42_ standards ranged from 1000 to 15.31 pg/ml. Aβ levels were then recalculated and expressed in nM based on a Mw of recombinant Aβ_1-42_ peptide ∼4514.1 D.

The levels of GFAP and S100B protein secreted into the culture medium were measured as previously described [31]. Briefly, the capturing, detecting and secondary antibodies for the GFAP ELISA were mouse monoclonal antibody (1:3000; Covance, Cleveland, OH, USA), rabbit polyclonal antibody (1:3000; Dako Cytomation) and AP-conjugated bovine anti-rabbit IgG (1:3000; Santa Cruz Biotechnology, Dallas, TX, USA), respectively. Purified GFAP protein from normal human brain (A86823H; Biodesign International, Memphis, TN, USA) was serial diluted (×2) and used as the standard protein, in concentrations ranging from 50 to 0.78 ng/ml. Mouse monoclonal S100 antibody (1:3000; Sigma-Aldrich), rabbit polyclonal S100 antibody (1:3000; Dako Cytomation) and AP-conjugated swine anti-rabbit IgG (1:3000; Dako Cytomation), respectively, were used as capture, detection and secondary antibodies in the S100B ELISA assay. The standard protein used was recombinant full-length protein corresponding to human S100B (ab54050; Abcam), in concentrations ranging from 1000 to 15.63 ng/ml (by two times serial dilution).

The levels of AChE protein and functional and total BuChE protein in the cultured medium were determined by using sandwich ELISA as described previously [32,33], with minor modifications involving the use of a 384-well plate from Nunc Maxisorb and the addition of 25 μl (in triplicate) of undiluted medium per well. The functional BuChE protein level was assessed as reported previously [13,14]. Briefly, after the completion of the sandwich ELISA, the plate was washed several times. Then 50 μl/well of a master-mix [containing butyrylthiocholine (5 mM) and the Ellman's reagent, DTNB (0.5 mM) in 50 mM phosphate buffer (pH 7.4)] was added. In this assay, the BuChE which were pre-adsorbed in the wells by the capturing primary antibody served as their own reporter enzyme system, quantifying only the proportion of the captured BuChE molecules that were enzymatically functional.

The concentration of ChAT protein concentration was measured by using a method we described earlier [17]. In brief, anti-human ChAT mouse monoclonal antibody (1:250; R&D Systems, Abingdon, UK), anti-human ChAT rabbit polyclonal antibody (1:2000; Abnova, Jhongli, Taiwan) and AP-Swine anti-rabbit (1:2000; Dako Cytomation) were used as capture, detection and secondary antibodies respectively. Triplicate samples of undiluted cell medium, 25 μl per well and a calibrated human plasma sample were used as standard. The end-point reactions for all ELISA measurements were monitored by using a microplate spectrophotometer reader (Tecan Infinite® M1000, Tecan Austria GmbH, Grödig, Austria) at 405 nm.

### AChE and BuChE activity measurements

AChE and BuChE activity were determined by using the modified Ellman's calorimetric assay described previously [32,33], and 25 μl of culture medium was added to each well, in triplicate samples.

### Multiplex immunoassays

The MSD human pro-inflammatory 9-plex ultrasensitive kit was used to quantify the cytokines in the cell medium secreted from microglia and neurospheres (Mesoscale Discovery, Gaithersburg, MD, USA). Twenty-five μl of cell medium from microglia or differentiating neurospheres was incubated in a total volume of 50 μl and the assay was performed according to the manufacturer's instructions. The MSD MULTI-SPOT plate was analysed on a Sector Imager 2400 (Mesoscale Discovery). Values from the medium collected from differentiating neurospheres were normalized to the baseline of the individual sample (at 11 DOD).

### Statistical analysis

Data are expressed as means ± SEM from three to four independent experiments. The unpaired Student's *t*-test was used for comparing two groups. anova followed by Dunnett's *post hoc* test was used to compare more than two groups by using GraphPad Prism 6.0 (GraphPad Software, Inc. La Jolla, CA, USA). Differences with *P* < 0.05 were considered significant.

## Results

### Differentiating neurospheres in culture exhibit immunogenic properties

**Fig. 1:**
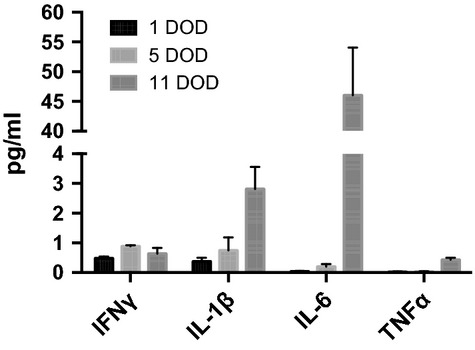
Neurospheres secrete pro-inflammatory cytokines. The secreted cell medium was collected at 1 DOD and 5 DOD and the secretion of the pro-inflammatory cytokines IFN-γ, IL-1β, IL-6 and TNF-α was measured by using a multiplex immunoassay. The values are expressed as mean ± SEM. DOD: days of differentiation; IFN: interferon; IL: interleukin; TNF: tumour necrosis factor.

**Fig. 2:**
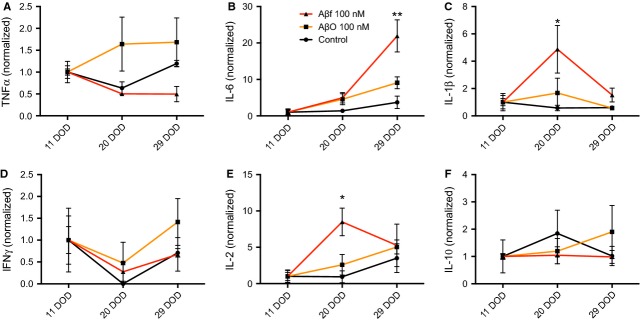
Fibrillar Aβ_1-42_ increases the secretion of cytokines from differentiating neurospheres. The secreted cell medium was collected at three time-points during neurosphere differentiation (11-29 DOD) and the release of the pro-inflammatory cytokines (**A**) TNF-α, (**B**) IL-6, (**C**) IL-1β, (**D**) IFN-γ, (**E**) IL-2 and (**F**) the anti-inflammatory cytokine IL-10 was measured by using a multiplex immunoassay. The values were normalized to baseline (11 DOD) and are expressed as means ± SEM (*P < 0.05, and **P < 0.01 compared to control). Aβ: amyloid beta; DOD: days of differentiation; Aβf: fibrillar Aβ; AβO: oligomeric Aβ; IFN: interferon; IL: interleukin; TNF: tumour necrosis factor.

Analysis of the culture medium from the differentiating neurospheres revealed that these cells secreted detectable levels of the cytokines interferon-γ (IFN-γ), IL-1β, IL-6 and TNF-α as early as 1 day of differentiation (1 DOD) (0.1–0.5 pg/ml, Fig. 1). Cell medium was changed 4 days later at 5 DOD, and higher levels of cytokines were measured, probably because of accumulation (Fig. 1). At 11 DOD, the secretion of the cytokines IL-2 and IL-10 also reached detectable levels (Fig. 2). At 11 DOD, the differentiating neurospheres consisted of a mixture of stem cells, neural progenitor cells, as well as differentiated neurons and glia [21,30]. The secretion of cytokines continued throughout differentiation up to 29 DOD (Fig. 2 A–D). The levels of IL-1β, IFN-γ, IL-2, TNF-α and IL-10 secreted from the neurospheres at 11 DOD were considerably lower (ranging from 0.5 to 2.8 pg/ml) than the levels secreted from microglia (ranging from 13.2 to 259.0 pg/ml), with the exception of IL-6 (50.8 pg/ml) that demonstrated similar levels to that secreted from microglia (46.1 pg/ml). Noteworthy, the levels of IL-6 are ∼35 pg/ml in plasma and 4.5 pg/ml in CSF of patients with AD [34].

### Fibrillar Aβ_1-42_ augments the secretion of pro-inflammatory cytokines from differentiating neurospheres

The step-wise protocol applied for the differentiation of neurospheres typically directs these cells towards a neuronal lineage with forebrain identity as we have previously demonstrated [18,21,30]. In the current study, the cytokine secretion profile from neurospheres during 29 DOD, following treatment with Aβf or AβO, relative to that of unstimulated (control) neurospheres, was compared as shown in Figure 2.

The secretion of IL-6 was significantly increased at 29 DOD (22.0 ± 4.4-fold) after Aβf treatment (100 nM), relative to control (*P* < 0.01, Fig. 2B). Aβf treatment also increased the secretion of IL-1β (4.9 ± 1.7-fold, *P* < 0.05, Fig. 2C) and IL-2 (8.5 ± 1.9-fold, *P* < 0.05, Fig. 2E) at 20 DOD relative to controls. In contrast, treatment with AβO (100 nM) did not alter the cytokine release profile in differentiating neurospheres (Fig. 2A–F).

### Fibrillar Aβ_1-42_ treatment increases the secretion of astroglial proteins in differentiating neurospheres

**Fig. 3:**
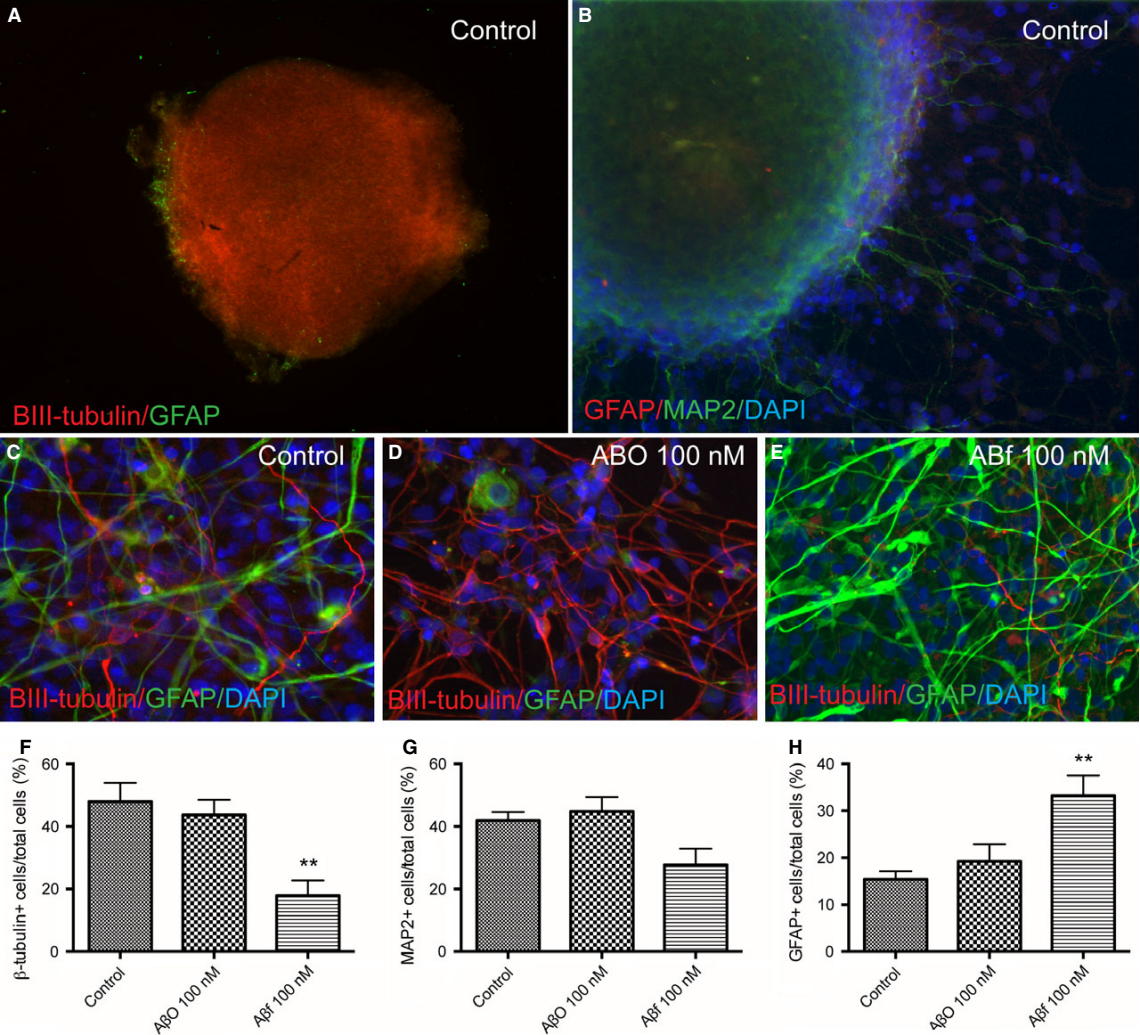
Fibrillar Aβ_1-42_ increases glial differentiation of neurospheres. Immunocytochemical staining of neuronal and glial markers (**A**) prior to Aβ exposure in neurospheres differentiated for 11 DOD, with immunoreactivity for the early neuronal marker βIII-tubulin (red) and the glial marker GFAP (green). (**B**) Unstimulated (control) neurospheres differentiated for 20 DOD, with immunoreactivity for the high molecular weight and low molecular weight neuronal marker MAP2 (green) and GFAP (red). Exposure of neurospheres to either AβO or Aβf differentiated for 27–29 DOD show immunoreactivity for βIII-tubulin (red) and GFAP (green) in (**C**) control cells, and the cells treated with (**D**) AβO and (**E**) Aβf preparations. The proportions of differentiated cells expressing βIII-tubulin, MAP2 or GFAP, respectively, following AβO or Aβf treatment are also shown (**F** and **G**). Values are expressed as the mean percentages of the total cells counted (DAPI immunoreactive cells) ± SEM from three independent experiments (>600 cells counted). **P < 0.01 compared to control. Aβ: amyloid beta; Aβf: fibrillar Aβ; GFAP: glial fibrillary acidic protein; MAP2: microtubule-associated protein 2; AβO: oligomeric Aβ.

Immunocytochemical and morphological examinations of the differentiating neurospheres were performed with the neuronal and astrocytic marker, βIII-tubulin and GFAP, respectively (Fig. 3). Prior to Aβ treatment (11 DOD), the differentiating neurospheres show abundant βIII-tubulin immunoreactivity, whereas GFAP immunoreactivity is mainly observed in the vicinity of the outer layer of the sphere (Fig. 3A). Whilst, unstimulated neurospheres differentiated for 20 DOD show immunoreactivity for high and low molecular weight MAP2, a marker for both developing and mature neurons (Fig. 3B). Typically at this stage, the cultured spheres preserve their spherical morphology, while a large numbers of cells differentiate and gradually migrate away from the spheres. These cells show neuronal morphology (Fig. 3B).

However, exposure of the neurospheres to Aβf for 29 DOD promoted the differentiation of astroglial cells, demonstrated by an increased proportion of cells that had migrated away from the sphere expressing the glial marker GFAP (33 ± 4%), and a reduction in the number of βIII-tubulin-expressing cells (18 ± 5%) compared with controls (16 ± 2% GFAP+ cells and 48 ± 6% βIII-tubulin+ cells, *P* < 0.01, Fig. 3C, E, F and H).

**Fig. 4:**
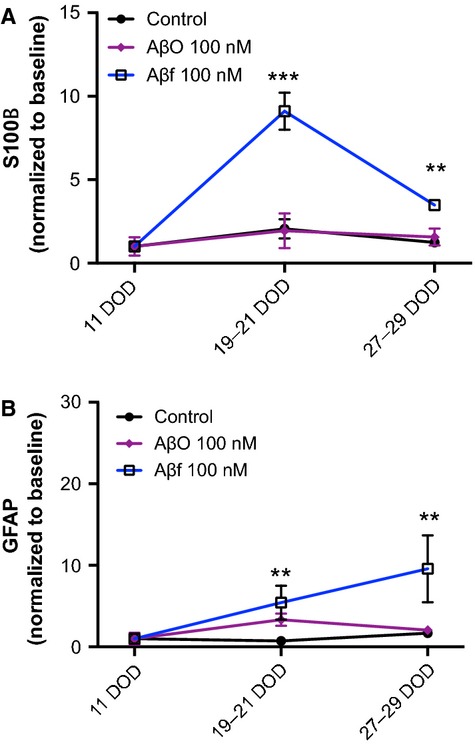
Fibrillar Aβ_1-42_ increases the expression of glial markers in differentiating neurospheres. Neurospheres were differentiated for 11 DOD, and subsequently treated once weekly with either AβO or Aβf. The cultured cell medium was collected and expression of the glial marker GFAP was measured at three time-points (11–29 DOD) by using sandwich ELISA. (**A**) The levels of the astrocytic cytokine S100B secreted from the neurospheres into the culture medium following exposure to AβO or Aβf. (**B**) Secretion levels of GFAP in the cultured medium from the neurospheres exposed to AβO or Aβf. Values were normalized to baseline (11 DOD) and are expressed as means ± SEM. **P < 0.01 and ***P < 0.001 compared to control. Aβ: amyloid beta; DOD: days of differentiation; AβO: oligomeric Aβ; Aβf: fibrillar Aβ; GFAP: glial fibrillary acidic protein.

In addition, the astroglial markers GFAP and S100B were secreted into the culture medium by differentiating neurospheres (Fig. 4A and B). S100B, in addition to its intracellular function, is regarded as an astrocytic secretory cytokine [35]. The differentiating neurospheres secreted S100B following treatment with 100 nM Aβf, but not with AβO (Fig. 4A). Aβf treatment induced 9.1 ± 1.1-fold increase in S100B levels at 19–21 DOD, and 3.5 ± 0.3-fold increase at 27–29 DOD relative to controls (*P* < 0.001, and *P* < 0.01, respectively, Fig. 4A).

Similarly, GFAP secretion from the differentiating neurospheres was significantly increased following treatment with Aβf, while no significant changes in secretion were observed after treatment with AβO (Fig. 4B). In comparison with controls, 100 nM Aβf increased the secretion of GFAP in the medium by 5.4 ± 2.1-fold at 19–21 DOD and 9.6 ± 4.1-fold at 27–29 DOD (*P* < 0.01, Fig. 4B).

The BrdU incorporation and the viability (MTS reduction) assays did not indicate that Aβ treatment affected the proliferation or the viability of the cells (Fig. S1A–F).

### Fibrillar Aβ_1-42_ decreases cytokine secretion from human primary microglia

As Aβf exposure enhanced the immunogenic profile of the differentiating neurospheres towards a glial phenotype, we investigated whether Aβ exposure would affect the cytokine secretion profile from pure human primary microglia cultures, which act as native immune cells in the CNS. As a positive control, we used LPS, for its known capacity to activate microglia.

**Fig. 5:**
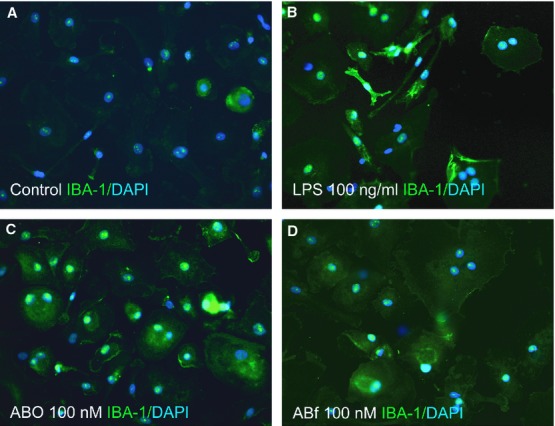
Fibrillar and oligomeric Aβ_1-42_ activate human microglia. Microglia were treated with Aβ_1-42_ peptides or LPS at the indicated concentrations every 48 hrs, for 14 days *in vitro*. The activated phenotype of the microglia was confirmed by immunocytochemical staining with IBA-1, a marker for activated microglia. (**A**) Immunoreactivity for IBA-1 (green) in the control (untreated); (**B**) immunoreactivity for LPS-treated microglia; (**C**) immunoreactivity for AβO_1-42_-exposed microglia; and (**D**) immunoreactivity for Aβf_1-42_-exposed microglia. Nuclei were stained with DAPI (blue). All micrographs are at 20 × magnification. A representative set of data is shown. Aβ: amyloid beta; Aβf: fibrillar Aβ; IBA-1: ionized calcium-binding adaptor molecule-1; LPS: lipopolysaccharide; AβO: oligomeric Aβ.

Microglia exposed to Aβf (100 nM), AβO (100 nM), or LPS (100 ng/ml) for 14 days were activated, as deduced by the increased expression of IBA-1 in treated cells (Fig. 5A–D). As expected, morphological examination of the LPS-exposed microglia showed the highest expression of IBA-1, while untreated cells (control) remained dormant as indicated by maintenance of their basal expression of IBA-1 (compare Fig. 5A and B). AβO and Aβf activated microglia to a lesser extent than those exposed to LPS (Fig. 5C and D).

**Fig. 6:**
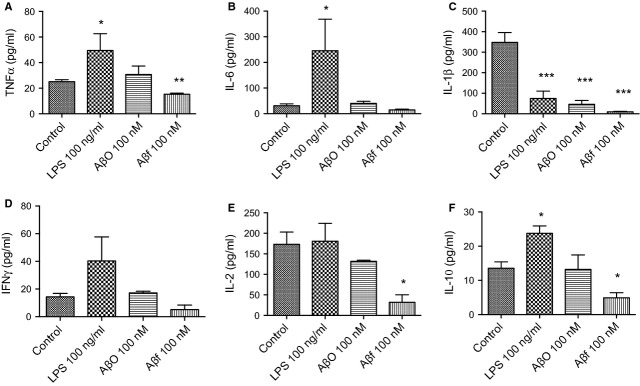
Fibrillar Aβ_1-42_ decreases the release of pro-inflammatory cytokines from microglia. The release of pro- and anti-inflammatory cytokines from human primary microglia was measured in the culture medium (conditioned medium) following 48-hr exposure to AβO, Aβf or LPS. The figure shows the levels of the pro-inflammatory cytokines (**A**) TNF-α, (**B**) IL-6, (**C**) IL-1β, (**D**) IFN-γ and (**E**) IL-2, and the anti-inflammatory cytokine (**F**) IL-10. Values are expressed as means ± SEM. *P < 0.05, **P < 0.01 and ***P < 0.001 compared to control. Aβ: amyloid beta; LPS: lipopolysaccharide; AβO: oligomeric Aβ; Aβf: fibrillar Aβ; TNF: tumour necrosis factor; IL: interleukin; IFN: interferon.

Examination of the cytokine secretion profile in the medium obtained from cultured microglia showed that compared with the control cells, LPS induced significant increases in the expression of TNF-α, IL-6 and IL-10 (*P* < 0.05, Fig. 6), while in contrast, a significant reduction in IL-1β levels (*P* < 0.001, Fig. 6C) was measured following 48 hrs of exposure.

Exposure of microglia to AβO for 48 hrs reduced the release of IL-1β by 87% compared to control (*P* < 0.001, Fig. 6C), while the secretion of the other cytokines assessed including IFN-γ, IL-2, IL-6, TNF-α and IL-10 (Fig. 6A–F) was not affected.

Interestingly, microglia treated with Aβf showed a decrease in the secretion of TNF-α (38% reduction, *P* < 0.01), IL-1β (97% reduction, *P* < 0.001), IL-2 (82% reduction, *P* < 0.05) and IL-10 (64% reduction, *P* < 0.05) compared with control (Fig. 6A–F).

Thus, with the exception of IL-1β, the exposure of microglia to LPS or Aβf resulted in two seemingly contrasting profiles of cytokine secretion. It is unlikely that this is because of toxicity induced by the treatments, as the incubation of microglia with LPS (100 ng/ml), or with pM–μM concentrations of Aβf or AβO did not induce nitric oxide production compared with control (data not shown). Although inducible nitric oxide synthase (iNOS) expression and nitric oxide production is well-established in rodent microglia, iNOS expression appears to be restricted to astrocytes in the human brain [36], which could explain the lack of nitric oxide production in the human microglia used herein. Alternatively, the secreted levels of nitric oxide were below the detection levels of the assay (Griess reagent system). Treatment with LPS, Aβf or AβO exposure for up to 5 days did not affect the viability of the cultured microglia, suggesting that the altered secretion of the cytokines was not because of the *in vitro* culture conditions (Fig. S2A and B).

### Neurospheres exposed to CM from Aβ_1-42_-treated microglia show impaired ability to differentiate into mature neurons

**Fig. 7:**
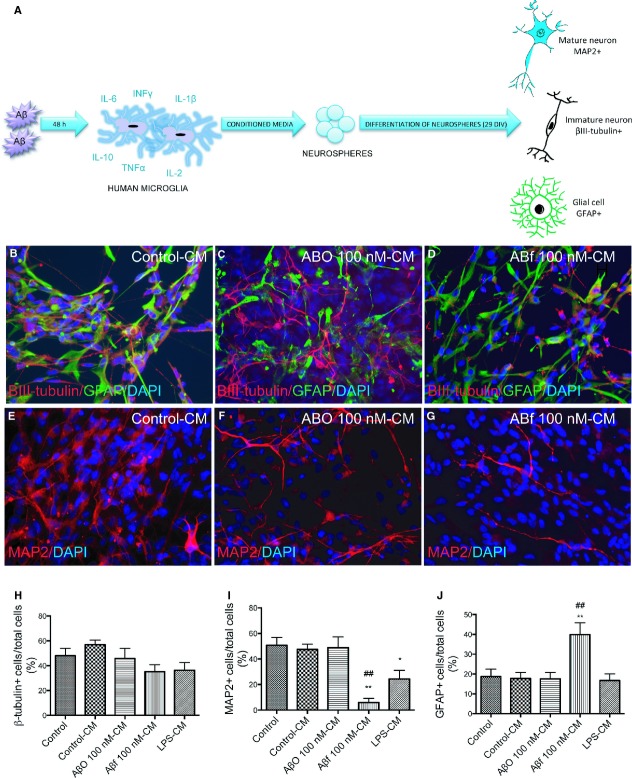
Fibrillar Aβ_1-42_ and conditioned medium from human microglia treated with fibrillar Aβ_1-42_ impair neurogenesis. (**A**) Schematic overview of the experimental setup. The CM was collected after exposing human microglia to LPS, AβO_1-42_ or Aβf_1-42_ for 48 hrs. Neurospheres were subsequently exposed to the CM (control-CM, LPS-CM, AβO-CM or Aβf-CM) and immunocytochemically stained with neuronal and glial markers. The figure shows immunoreactivity for the early neuronal marker βIII-tubulin (red) and the glial marker GFAP (green) in stem cells treated with (**B**) control-CM, (**C**) AβO-CM and (**D**) Aβf-CM. Immunoreactivity for the late neuronal marker MAP2 (red) following exposure to (**E**) control-CM, (**F**) AβO-CM and (**G**) Aβf-CM is also shown. Nuclei were stained with DAPI (blue). All micrographs are at 20 × magnification. (**H**–**J**) The proportion of cells expressing βIII-tubulin, MAP2 or GFAP, respectively, following control-CM, LPS-CM, AβO-CM or Aβf-CM treatment. Values are expressed as the mean percentages of the total cells counted (DAPI immunoreactive cells) ± SEM from three independent experiments (>600 cells counted). *P < 0.05, and **P < 0.01 compared to control and ^##^*P* < 0.01 compared to control-CM. Aβ: amyloid beta; CM: conditioned medium; AβO: oligomeric Aβ; Aβf: fibrillar Aβ; GFAP: glial fibrillary acidic protein; LPS: lipopolysaccharide; MAP2: microtubule-associated protein 2.

To determine whether the altered secretion of cytokines from microglia observed after Aβf and LPS exposure could influence the differentiation of the neurospheres, we treated the neurospheres with CM for 29 DOD and thereafter performed immunocytochemical staining with markers for immature and mature neurons, as well as for glia cells. A schematic overview is illustrated in Figure 7A.

The addition of control-CM or AβO-CM did not influence the numbers of βIII-tubulin+, MAP2+ or GFAP+ cells compared with controls (Fig. 7 B–J). The addition of Aβf-CM, however, impaired neuronal maturation, as the number of MAP2+ cells was lower with Aβf-CM (6 ± 3%) compared with the differentiation of untreated neurospheres (51 ± 6%, *P* < 0.01), or to neurospheres treated with control-CM (48 ± 4%, *P* < 0.01, Fig. 7I). The number of GFAP+ cells was increased following treatment with Aβf-CM (40 ± 3%) in comparison with the untreated controls (19 ± 4%, *P* < 0.01) or with the control-CM (18 ± 3%, *P* < 0.01, Fig. 7J). Interestingly, treatment with the LPS-CM from the microglia, which contained extensive secreted levels of pro-inflammatory cytokines, resulted in a decreased number of MAP2+ cells (24 ± 7%) compared with controls (51 ± 6%, *P* < 0.05, Fig. 7I).

Irrespective of the conditioning factors, treatment with CM generally increased the proliferation rate of the neurospheres, compared with that of the untreated controls (Fig. S1C). This could possibly be attributed to the foetal calf serum that was present in the CM, but not in the culture medium used for the control cells.

The assessment of the cells viability with the MTS reduction assay indicated that treatment with the CM also increased the metabolic rate of the differentiating neurospheres. However, this was again only compared with the untreated controls (Fig. S1F). The MTS reduction assay demonstrated no changes when control-CM was compared with Aβ-CM or LPS-CM. Thus, although this supports the notion regarding the effect of foetal calf serum on the proliferation rate of the neurospheres (Fig. S1F), it is unlikely that foetal calf serum affected the differentiation of neurospheres in a particular direction, as the control-CM also contained this serum.

To control any carry-over effect of Aβf in the Aβf-CM, the concentration of Aβ was quantified in all microglia CM. In control-CM, no Aβ_1-42_ peptides were detected, indicating that production and release of any endogenous Aβ by the primary microglia were too low (if occurred) to affect the results. In the Aβf-CM, the concentration of Aβ_1-42_ was 100 ± 10 pM. Considering that this Aβf-CM was used as 1:2 dilution of NPM for the treatment of differentiating neurospheres, the overall amount of Aβ carried over was 50 ± 5 pM, which is about 2000-fold less than the starting 100 nM Aβf concentration.

### Fibrillar Aβ_1-42_ alters the secretion of the ACh-regulating enzymes, ChAT and BuChE from differentiating neurospheres

The cholinergic signalling molecule ACh has previously been shown to exert immune-regulatory and anti-inflammatory effects [37]. In addition, functional variability in the ACh-degrading capacity of BuChE, which may be induced by various factors, shows a strong relationship with the intrathecal cytokine and astroglial profiles in AD patients [34]. We have also recently shown that hES cells and astrocytes isolated from human brain produce and release the ACh-synthetizing enzyme, ChAT into the culture medium [17].

Given that the current results suggest that Aβf and Aβf-CM direct the differentiation of neurospheres into astroglia, we investigated whether the release of ChAT, AChE and BuChE from neurospheres during their differentiation is related to their intrinsic cytokine secretion profile, and whether this is altered in the presence of Aβf peptides.

**Fig. 8:**
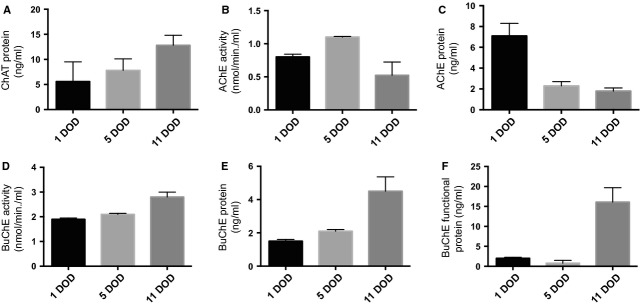
Neurospheres secrete the cholinergic enzymes AChE, BuChE and ChAT. Neurospheres were cultured for 1 DOD and 5 DOD and the culture cell medium was collected, and the expression of ChAT, AChE and BuChE was measured by ELISA. The figure shows the levels of (**A**) ChAT protein, (**B**) AChE activity, (**C**) AChE protein, (**D**) BuChE activity, (**E**) BuChE protein and (**F**) functional BuChE protein released in the culture medium. Values are given as mean ± SEM. BuChE: butyrylcholinesterase; AChE: acetylcholine esterase; ChAT: choline acetyltransferase; DOD: days of differentiation.

The protein levels of these cholinergic enzymes secreted into the culture medium by neurospheres were detectable already at 1 DOD and 5 DOD (Fig. 8). The levels at 1 DOD were: ChAT 5.6 ± 3.9 ng/ml, AChE 7.1 ± 1.0 ng/ml and BuChE 1.5 ± 0.1. At the onset of Aβ treatment at 11 DOD, the levels of ChAT and AChE had increased (ChAT 12.8 ± 2.0 ng/ml and AChE 1.8 ± 0.3 ng/ml), whereas BuChE decreased (BuChE 4.5 ± 0.8 ng/ml). The average activity at 1 DOD for AChE was 0.8 ± 0.04 nmol/min./ml and for BuChE 1.9 ± 0.05 nmol/min./ml (Fig. 8B and D). At 11 DOD, the levels were comparable, and the average activity measured for AChE and BuChE was 0.5 ± 0.2 nmol/min./ml and 2.8 ± 0.2 nmol/min./ml respectively.

**Fig. 9:**
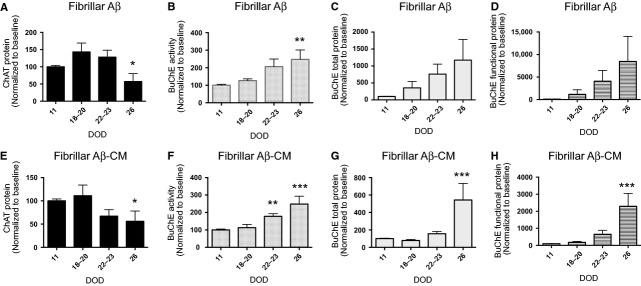
Fibrillar Aβ_1-42_ treatment decreases ChAT protein expression and increases BuChE activity and protein expression in differentiating neurospheres. Neurospheres were differentiated for 11 DOD and subsequently treated once weekly with 100 nM Aβf, or with the CM obtained by exposing microglia to 100 nM Aβf (Aβf-CM). The cultured cell medium was collected and the expression of ChAT was measured by sandwich ELISA at four time-points (11–26 DOD). The figure shows the levels of (**A**) ChAT protein, (**B**) BuChE activity, (**C**) total BuChE protein and (**D**) functional BuChE protein released in the culture medium by neurospheres treated with Aβf. The levels of (**E**) ChAT protein, (**F**) BuChE activity, (**G**) total BuChE protein and (**H**) functional BuChE protein released in the culture medium by neurospheres treated with Aβf-CM. *P < 0.05, **P < 0.01 and ***P < 0.001 compared to control (11 DOD). The values are normalized to baseline at 11 DOD and given as means ± SEM. Aβ: amyloid beta; BuChE: butyrylcholinesterase; ChAT: choline acetyltransferase; CM: conditioned medium; DOD: days of differentiation; Aβf: fibrillar Aβ.

Treatment with Aβf increased ChAT secretion from the neurospheres at 18–20 DOD, while a steady decrease in ChAT secretion was measured during the remaining course of differentiation (Fig. 9A, *P* < 0.05). A different pattern was observed for BuChE. Aβf treatment induced gradual increases in BuChE activity (Fig. 9B, *P* < 0.01) and consistent increases in the levels of BuChE protein secreted into the culture medium throughout the course of neurosphere differentiation (26 DOD) compared with baseline (Fig. 9C and D).

Exposure of the neurospheres to Aβf-CM induced a significant decrease in ChAT secretion (Fig. 9E, *P* < 0.05), followed by a concomitant increase in BuChE activity and protein during differentiation, compared with baseline (Fig. 9F–H, *P* < 0.001). The secretion of ChAT and BuChE from differentiating neurospheres did not change over time following exposure to AβO (data not shown). Furthermore, a comparison between the levels of functional BuChE (Fig. 9D and H) and the levels of total BuChE protein (Fig. 9C and G) suggested that Aβf did not merely increase the release of BuChE from the differentiating neurospheres, but also induced a hyperactive phenotype of BuChE, a phenomenon that has also been reported in CSF of AD patients [13,14,38].

The microglia either did not release ChAT, or the levels of ChAT released from microglia into the culture medium were below the detection levels of our sandwich ELISA.

## Discussion

This study demonstrates that Aβ, in particular Aβf or Aβ protofibrils, causes a change in the microenvironment that favours the differentiation of hES-derived neurospheres into glial cells. Analysis of the profiles of cytokines secreted from the differentiating neurospheres further indicated that the physiological levels of the cytokines combined may be an important determinant for whether neurogenesis or gliogenesis occurs in neural stem-cell/progenitor cell populations.

### Fibrillar Aβ increases cytokine secretion from differentiating neurospheres and induces astroglial differentiation

The current study revealed that differentiating neurospheres exhibit an intrinsic profile of cytokine secretion, suggesting that the basal physiological concentrations of cytokines may exert a modulatory role on neurogenesis by shaping the neurogenic niche. Previous studies have demonstrated that stem cells are able to respond to cytokine signalling [23,24]. A study on hES cell-derived neurospheres have shown that they secrete the anti-inflammatory cytokines IL-10 and TGF-β [26]. Our findings are in line with these previous findings, but in addition show that neurospheres release detectable levels of IL-1β, TNF-α, IFN-γ and IL-6 as early as 1 day of differentiation, suggesting that stem cells may be able to initiate a pro-inflammatory process. Overall, these findings may either reflect the intrinsic immunogenic properties or the influence of cytokines on stem cells' neurogenic properties. The immunogenic properties of neurospheres may have further implications for both the survival and integration of grafted stem cells in *in vivo* models, as well as potentially initiating a graft-versus-host reaction. We propose that stem cell/progenitor cells possessing immunomodulatory properties may influence neuroplasticity and regenerative mechanisms in the brain, especially during AD pathogenesis.

We found that the secreted levels of IL-6 at 11 DOD of the differentiating neurospheres into the culture medium reached comparable concentrations with the IL-6 levels found in the plasma, but much higher than in CSF of patients with AD [34]. Although such a comparison should be considered with due caution, this suggests that IL-6 might play a role in the regulating mechanisms of stem-cell differentiation, where divergences from the basal physiological secretions could be unfavourable for neurogenesis. For instance, we found that exposure of the differentiating neurospheres to Aβf increased the secretion of IL-6 over 20-fold. This coincided with reduced neuronal differentiation of the neurospheres, but with increased numbers of stem cell-derived glial cells. Our findings are in line with a report showing that overexpression of IL-6 from astroglial cells can restrain adult neurogenesis [39]. Nevertheless, the outcome of the IL-6 effect seems to require additional factors or cytokines, as IL-6 also enhances neurogenesis of human neural precursor cells *in vitro* [24], and *in vivo* studies in IL-6 knock-out mice show compromised neurogenesis [40].

Morphological microscopy analyses showed that prior to Aβ treatment (11 DOD), the differentiating neurospheres expressed abundant βIII-tubulin, indicative of a high proportion of immature neurons. However, βIII-tubulin is expressed in both glial and neuronal precursors [41], indicating presence of abundant progenitor cells in the centre of the sphere. Following Aβf exposure, the numbers of differentiated glial (GFAP+) cells were, however, increased. We also demonstrated a transient increase in the levels of S100B, an astrocytic secretory protein with cytokine-like properties, while the levels of GFAP, a cytoskeleton protein, steadily increased throughout the course of differentiation. Altogether, these findings suggest that the released cytokines either drive the differentiation of the neurosphere towards gliogenesis or are a consequence of the increased gliogenesis induced by Aβf treatment. The transient nature of the increases in IL-1β, IL-2 and S100B, following Aβf treatment, additionally suggest that these signalling molecules may be involved in mediating fate-commitment of differentiating neurospheres. This suggests a mechanism in which Aβf stimulates propagation of immunogenic astroglial cells to secrete soluble factors during their differentiation, disrupting maturation of differentiating neuronal precursor cells. We hence suggest that the Aβ-induced pattern of cytokine release is a context-dependent phenomenon to directly or indirectly fine-tune the neurogenic niche, which may ultimately alter neuroplasticity and regeneration.

However, further studies are needed to investigate whether the increased cytokine secretion from differentiating neurospheres reflects the increased number of generated astroglial cells, or whether this is the consequence of an intrinsic immunogenic response of differentiating stem cells to Aβf exposure, as an attempt to neutralize their microenvironment by producing and/or recruiting more astroglial cells. Nonetheless, we showed that the cytokine secretion over time showed little temporal changes in the untreated (control) neurospheres, which normally generate high numbers of neurons [18,30], making it less likely that the increased levels of cytokines in the cultured medium of the Aβf-treated neurosphere neurons are the primary source of cytokine secretion. Indeed, CSF, as well as *in vivo* imaging studies, suggests that maintenance of a heightened functional status of astroglial cells may be protective, and follows an inverted U-shape dynamic in the continuum of AD [34,42].

### Aβf-CM from microglia impairs neural differentiation

Both an increased activation of microglia and microglia senescence are implicated in AD pathogenesis [1,35], as well as in non-neural regulation of neurogenesis [9]. Thus, we were interested to study whether Aβ alters the native immunogenic responses of human microglia in a similar fashion as the classical immunogenic inducer, LPS, and whether these changes influence neurogenesis. We found that reduced secretion of the cytokines from microglia following exposure to Aβf coincided with impaired neural differentiation of the neurospheres. Moreover, we demonstrate that conditioned medium from Aβ-treated microglia increased the number of GFAP-positive cells, but decreased the number of MAP2-positive cells during differentiation of the neurosphere cultures. However, the expression of the neuronal marker βIII-tubulin remained virtually unchanged. βIII-tubulin is considered as one of the earliest neuronal markers, which is also expressed in nestin-positive and GFAP-positive cells [41]. In contrast, the high molecular weight MAP2 expression is found only in neurons that have commenced dendrogenesis [43]. Thus, the observed discrepancy between expression levels of βIII-tubulin and MAP2 may reflect compromised *in vitro* maturation of the newly generated neurons, which in turn may suggest that suppressing a basal physiological cytokine secretion from microglia may alter endogenous neurogenesis in favour of gliogenesis. This is in line with previous reports showing that different concentrations of cytokines may exert diverse effects that influence neurogenesis. For instance, low levels of TNF-α enhance proliferation, whereas supraphysiological TNF-α levels inhibit proliferation and neuronal differentiation of mice neural progenitors [44]. Thus, basal secretion of cytokines may be important for normal cell physiology, possibly reflecting the dual function of the innate immune system in modulating cell genesis and repair. Alternatively or additionally, these findings may point at an unidentified factor(s) in the Aβf-CM that compromises the neuronal differentiation of the neurospheres.

As expected, LPS stimulation of microglia increased the secretion of the pro-inflammatory cytokines. Moreover, the CM from LPS-stimulated microglia, containing high levels of cytokines, impaired the ability of the neurospheres to differentiate into mature neurons, as evidenced by the decreased immunoreactivity of the mature neuronal marker MAP2. These findings are consistent with observations by other groups studying hippocampal neurogenesis in rats [10,11], but to the best of our knowledge, our study is the first demonstrating this phenomenon in human cells. Nonetheless, we found a significant reduction in the secretion of IL-1β, following LPS, AβO and Aβf exposures. Chronic LPS exposure seems to down-regulate IL-1β mRNA expression in microglia [45]. In the context of neurogenesis, this is an important observation and is consistent with the proposed neurotrophic effects of IL-1 [46].

Noteworthy, exposure of microglia to Aβ and LPS unexpectedly triggered fundamentally different immunogenic responses from the cultured human microglia in our study. This suggests that LPS-induced inflammation may not be a suitable model for studying inflammatory responses in the course of AD.

We also found that exposure of microglia to AβO did not influence secretion of the cytokines, other than down-regulating IL-1β. Consequently, it is possible that AβO is not one of the key components regulating the inflammatory component of microglia or differentiating neurospheres. In addition, this finding is contrary to earlier observations in studies, conducted on murine cell lines, where AβO increased secretion of pro-inflammatory cytokines from mouse microglia, and the CM from AβO-treated mouse microglia induced neurotoxicity in mouse primary hippocampal neurons [5]. These discrepancies may hence reflect specific responses for different species, highlighting the importance of using human cell lines.

### Fibrillar Aβ causes a shift in the ACh-regulatory machinery

Cholinergic signalling in the peripheral circulation exerts a well-established immune-regulatory effect on systemic inflammatory responses *via* the action of ACh putatively on the α7 nAChRs present on immune cells [37,47].Emerging evidence also suggests a similar regulatory contribution of cholinergic signalling on the functional status of cholinoceptive astroglial cells in the CNS [17,34,48,49]. For instance, we have recently demonstrated that ChAT is actively secreted by human brain primary astrocytes and that soluble ChAT maintains a certain level of extrasynaptic ACh in the presence of fully active and naturally occurring levels of the ACh-hydrolysing enzymes AChE and BuChE [17]. Thus, it is conceivable that imbalanced cholinergic signalling and disruption of the normal anti-inflammatory signalling of ACh could also alter the properties of neural progenitors/stem cells residing in the neurogenic niche with consequences on neuronal plasticity and endogenous regeneration in the adult brain. To gain some insights in these regards, we studied the impact of exposure to Aβf and Aβf-CM on cholinergic signalling in microglia and differentiating neurospheres.

First, we found that differentiating neurospheres secrete ChAT, the enzyme responsible for biosynthesis of ACh as well as the ACh-degrading enzymes AChE and BuChE as early as 1 DOD. At 11 DOD, just prior to exposure of the cells to Aβ, the levels of these enzymes in the culture medium reached comparable levels with those in human CSF [17]. This high basal secretion of all three cholinergic enzymes involved in the homoeostasis of ACh implies a prominent role for cholinergic signalling in the developmental biology of stem cells, which is in line with previous reports [50].

Next, we found that both Aβf and Aβf-CM caused a time-dependent reduction in the release of ChAT, levels of which peaked at 19–20 DOD, but then gradually decreased to as low as one-fourth of its peak *in vitro* levels, while the secreted levels of the ACh-degrading enzyme BuChE showed a highly significant increase. Thus, prolonged exposure of the differentiating neurospheres to both Aβf and Aβf-CM caused a pronounced shift in the ACh-regulatory machinery that favoured maintenance of low levels of ACh. Given that ACh is expected to exert suppressive, anti-inflammatory effects on immunogenic cells such as the cholinoceptive astroglial cells, these gradual Aβ-directed changes in the secretion levels of these cholinergic enzymes seem synchronized to generate a microenvironment specifically promoting astrogliogenesis, as suggested by the microscopic analysis.

Studies of CSF samples from patients with AD are consistent with these observations and suggest that Aβ allosterically potentiates the ACh-hydrolysing capacity of BuChE [16], particularly in the presence of high levels of apolipoprotein E [14–16,38]. Here, we showed that Aβ affects the levels of cholinergic enzymes ChAT and BuChE in two ways. Firstly, Aβf altered the protein expression and release levels of ChAT and BuChE. Secondly, Aβf altered the intrinsic enzymatic rate of BuChE to a hyperactive phenotype as the levels of the functional BuChE protein were much higher than the total levels of BuChE protein in the cultured medium.

Altogether, these observations support the suggested mechanisms for the early cholinergic dysfunction in AD [16]. The current observations are also in agreement with reports, suggesting that different Aβ assemblies in AD brains are associated with reduced ChAT activity and reduced numbers of nAChRs [51], but increased BuChE activity [52,53].

Nevertheless, it should be noted that the current study cannot exclude the possibility that the changes in the cholinergic enzymes are not simply a consequence, rather than a driving force of the increased astrogliosis observed after Aβf or Aβf-CM treatment paradigms. Future studies using selective BuChE inhibitors are required to provide a definitive answer to this question. Another limitation of the study is that a small proportion of the Aβf was carried over from the Aβf-CM, which could not be avoided without extensive manipulation of Aβf-CM. However, the carried-over amount of Aβf was over 2000-fold less than the starting 100 nM Aβf concentration. It is noteworthy that treatment with 10 nM Aβf did not show any significant effect on the differentiating neurospheres (data not shown), suggesting that any effect related to the carried-over levels of 50 pM Aβ should be negligible on the current results. Finally, it should also be noted that primary microglia cell cultures may, under the *in vitro* conditions, acquire amoeboid morphology, which are not necessarily representing an *in vivo* functional status [54,55]. Such discrepancies may account for aberrant findings in the current study, for instance, the decrease in IL-1β secretion.

In conclusion, we have shown that differentiating neurospheres secrete various cytokines and cholinergic enzymes involved in the homoeostasis of ACh signalling as early as 24 hrs of differentiation. Our observations lead us to propose a mechanism whereby Aβf decreases neurogenesis by promoting a microenvironment favouring hypo-cholinergic signalling and gliogenesis. These novel findings advance our understanding of the basic regulatory processes involved in cell genesis, and warrant further studies to deduce the involvement of receptors and down-stream signalling mechanisms.

## Supporting information

**Figure S1** Cell proliferation and cell viability of cultured neurospheres treated with fibrillar or oligomeric Aβ_1-42_.

**Figure S2** Aβ_1-42_ exposure does not affect the viability of human primary microglia in culture.
